# A prognostic nomogram for predicting overall survival in colorectal mucinous adenocarcinoma patients based on the SEER database

**DOI:** 10.17305/bjbms.2022.8297

**Published:** 2023-05-01

**Authors:** Qian Wu, Suqin Zhang, Huan Wang, Yifei Zeng, Wei Yang, Wenjun Pan, Guodai Hong, Wenbin Gao

**Affiliations:** 1 Department of Oncology, Shenzhen Luohu People’s Hospital, Shenzhen, China

**Keywords:** Colorectal cancer (CRC), mucinous adenocarcinoma, nomogram, overall survival (OS), Surveillance, Epidemiology and End Result (SEER) program

## Abstract

A nomogram was constructed to predict the survival of patients with colorectal mucinous adenocarcinoma based on data extracted from the Surveillance, Epidemiology and End Result (SEER) database. Data collected between 2010 and 2018 were obtained from the SEER database. The log-rank test and multivariate Cox regression were performed to identify the independent prognostic factors for overall survival, which were further used to construct a nomogram model to predict 1-, 3-, and 5-year overall survival. In total, 10,846 patients diagnosed with colorectal mucinous adenocarcinoma were enrolled in the study. The following 11 variables were associated with survival and were further incorporated into the nomogram: age at diagnosis, primary site, grade, tumour size, lymph node dissection, T stage, N stage, M stage, surgery for primary site, chemotherapy, and household income. The concordance index (C-index) value was 0.725 (95% confidence interval 0.716–0.734), and the receiver operating characteristic curves and calibration curves showed satisfactory predictive accuracy. Both the C-index and time-independent area under the curve values were greater than those of the American Joint Committee on Cancer 7th TNM classification system (both *P* < 0.001). In the validation group, the results were consistent with those of the training group, with a C-index value of 0.726 (95% confidence interval 0.713–0.739). This study constructed a practical nomogram to predict 1-, 3-, and 5-year OS for patients with colorectal mucinous adenocarcinoma based on the SEER data.

## Introduction

Colorectal cancer (CRC) is the third most common cancer and the second leading cause of cancer-related death worldwide [1]. The majority of CRC cases are adenocarcinomas, accounting for approximately 85% of cases. Mucinous adenocarcinoma (MAC) is a less common subtype that accounts for 8%–19% of CRC cases and is defined by the World Health Organization (WHO) as the presence of extracellular mucin in >50% of the tumor area [2, 3]. Many studies have demonstrated the distinct clinical and pathological features of MAC, which is regarded as being more advanced at diagnosis and has a poorer prognosis than nonmucinous adenocarcinoma (NMAC) [4, 5].

Despite advances in treatment strategies, such as targeted therapy and immunotherapy, the 5-year survival of locally advanced and metastatic CRC patients is still unsatisfactory [6]. Effective methods for precision medicine and prognosis prediction are in high demand. Currently, the most prevalent method for prognosis and treatment direction is the tumour-node-metastasis (TNM) staging system, which is classified mainly by the depth of primary tumour invasion (T), numbers of regional lymph node metastases (N), and distant metastasis (M) [7–9]. However, many other clinical, pathologic, and economic factors that have been proven to be related to survival, such as age, race, tumour size, histological subtype, grade, household income, and information about treatment, such as surgery, radiotherapy, and chemotherapy, are not included in this system, influencing the accuracy of its predictive ability [5, 10, 11]. Thus, it is imperative to explore a novel program that can comprise all prognostic factors to predict the outcomes of cancer patients.

A nomogram is a simple visual predictive model that is widely used for prognosis prediction. It is more comprehensive in terms of the inclusion of all effective prognostic factors and more intuitive because the estimated survival of each patient can be conveniently calculated by combining the score of each parameter and matching the corresponding percentage [12]. To the best of our knowledge, there is currently no nomogram specialized for patients with colorectal MAC. Thus, the aim of this study was to establish a nomogram to predict the survival of colorectal MAC patients based on data extracted from the Surveillance, Epidemiology and End Result (SEER) database.

## Materials and methods

### Data source and patient selection

Eligible patients were extracted from the database “Incidence-SEER Research Data, 18 Registries, Nov 2020 Sub(2000–2018)” using the SEER*Stat program (v8.4.0). The inclusion criteria were as follows: (1) patients who were diagnosed with primary colorectal adenocarcinoma according to the third version of the International Classification of Disease for Oncology (ICD-O-3) and (2) patients who had histologically confirmed MAC subtype (ICD-O-3 coded as 8480/8481). The exclusion criteria were as follows: (1) patients with more than one primary cancer; (2) patients diagnosed by clinical criteria or based on autopsy or the death certificate; (3) patients with incomplete survival information; (4) patients with a follow-up of less than 1 month; and (5) patients with incomplete clinicopathological and treatment information (including primary site, histological type, grade, tumour size, lymph node dissection number, American Joint Committee on Cancer [AJCC] 7th TNM stage, surgery, radiotherapy, chemotherapy, and median household income). Eligible patients were randomly divided into a training group and a validation group in a 70:30 ratio.

### Variables and endpoints

In this study, 14 variables were collected from the database, including sex, age at diagnosis, race, primary site, grade, tumour size, lymph node dissection, T stage, N stage, M stage, surgery, radiotherapy, chemotherapy, and median household income. All tumours were staged according to the TNM staging system of the AJCC (7th version, 2009). The primary tumour site was divided into the caecum-ascending colon (including the appendix, caecum, ascending colon, and hepatic flexure), transverse colon, descending colon-sigmoid (including the descending colon, sigmoid colon, and splenic flexure), and rectum. For continuous variables, patients were divided into two groups, with the age of 65 years as the cutoff. The cutoff value for tumour size was 3 cm. The endpoint of this study was overall survival (OS), which was defined as the duration between diagnosis and death due to all causes.

### Ethical statement

All patient data were obtained from the SEER database, which records cancer data for approximately 30% of the American population across different regions [13]. A SEER Research Data Agreement (No. 12068-Nov2021) was signed for data acquisition. Given that the data were publicly accessible and deidentified, patient informed consent was not needed, and no approval from an ethics committee was demanded. This research was performed in accordance with the Declaration of Helsinki.

### Statistical analysis

#### Nomogram construction

Categorical variables are shown as proportions and frequencies and were compared by the Chi-square test or Fisher’s exact test. The associations between each variable and survival were first evaluated with univariate analysis using the log-rank test. Variables with a *P* value ≤ 0.1 during univariate analysis were further examined by multivariate backward stepwise Cox proportional hazard regression analysis. Statistically significant variables in the multivariate Cox regression analysis (*P* ≤ 0.05) were determined to be independent prognostic factors to predict the survival outcome. Additionally, variance inflation factor examinations of the effective prognostic factors were evaluated to exclude multicollinearity problems. Then, these selected factors were used to establish a nomogram model to predict 1-, 3-, and 5-year OS in the training and validation groups performed by the rms package in R (version 4.1.2).

#### Nomogram validation

The performance of the nomogram was evaluated by detecting its discrimination and calibration abilities both internally (in the training group) and externally (in the validation group). The bootstrapping resampling approach (1000 repetitions) was applied to interval validation. The concordance index (C-index) and the receiver operating characteristic (ROC) curve were used to validate discrimination performance. A higher C-index value and a larger area under the curve (AUC) in the ROC curve represented better discrimination ability. In addition, we calculated the C-index and the ROC curve using the AJCC 7th TNM classification system and then compared the results with our nomogram to identify differences. The calibration curves were used to evaluate the calibration ability of the nomogram. A 45-degree plot represented an optimal model. All statistical analyses were conducted using SPSS 26.0 (SPSS Inc., Armonk, NY, USA) and R (version 4.1.2, www.r-project.org). A difference of *P* < 0.05 (two-tailed) was considered statistically significant.

## Results

### Patient characteristics

**Figure 1.**
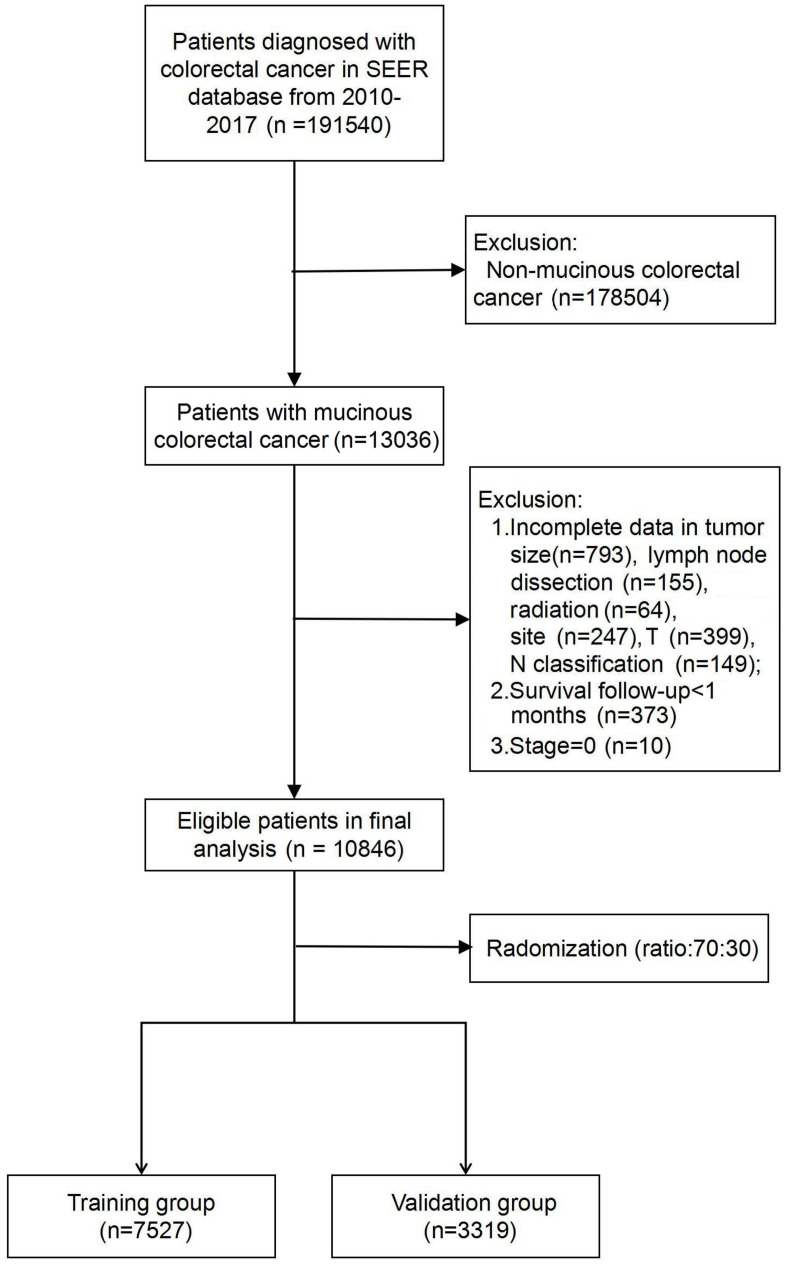
Flowchart of patient selection.

In total, 10,846 patients diagnosed with colorectal MAC were enrolled in the study and were assigned to the training group (*n* ═ 7527) or the validation group (*n* ═ 3319). A detailed flowchart of patient selection is presented in Figure 1. Among all the included patients, the majority were female (*n* ═ 5639, 52.0%), aged ≥ 65 years (*n* ═ 6158, 56.8%) and white (*n* ═ 8793, 81.1%). A total of 71.7% of patients had a household income higher than 75,000 dollars. The most common primary site was the right colon (*n* ═ 6645, 61.3%), followed by the left colon (*n* ═ 2324, 21.4%), rectum (*n* ═ 983, 9.1%), and transverse colon (*n* ═ 894, 8.2%). The tumour grade proportions of all patients were well (*n* ═ 1374, 12.7%), moderate (*n* ═ 7171, 66.1%), poor (*n* ═ 1832, 16.9%), and undifferentiated (*n* ═ 469, 4.3%). More than 80% of the patients had a tumour larger than 3 cm (*n* ═ 8958, 82.6%). Stages T1-4 accounted for 3.8%, 11.2%, 55.8%, and 29.2% of the population, respectively. Regarding the treatment methods, only 1.2% of the patients did not undergo surgery, and 92.7% of the patients had more than 4 lymph node dissections. Radiotherapy and chemotherapy were administered to 9.3% and 44.8% of the patients, respectively. The 1-, 3-, and 5-year OS rates of all enrolled patients were 87.4%, 68.3%, and 57.2%, respectively. The detailed clinicopathological features of the two groups are listed in Table 1. There were no significant differences between the training and the validation group.

### Variable selection and nomogram construction

**Figure 2.**
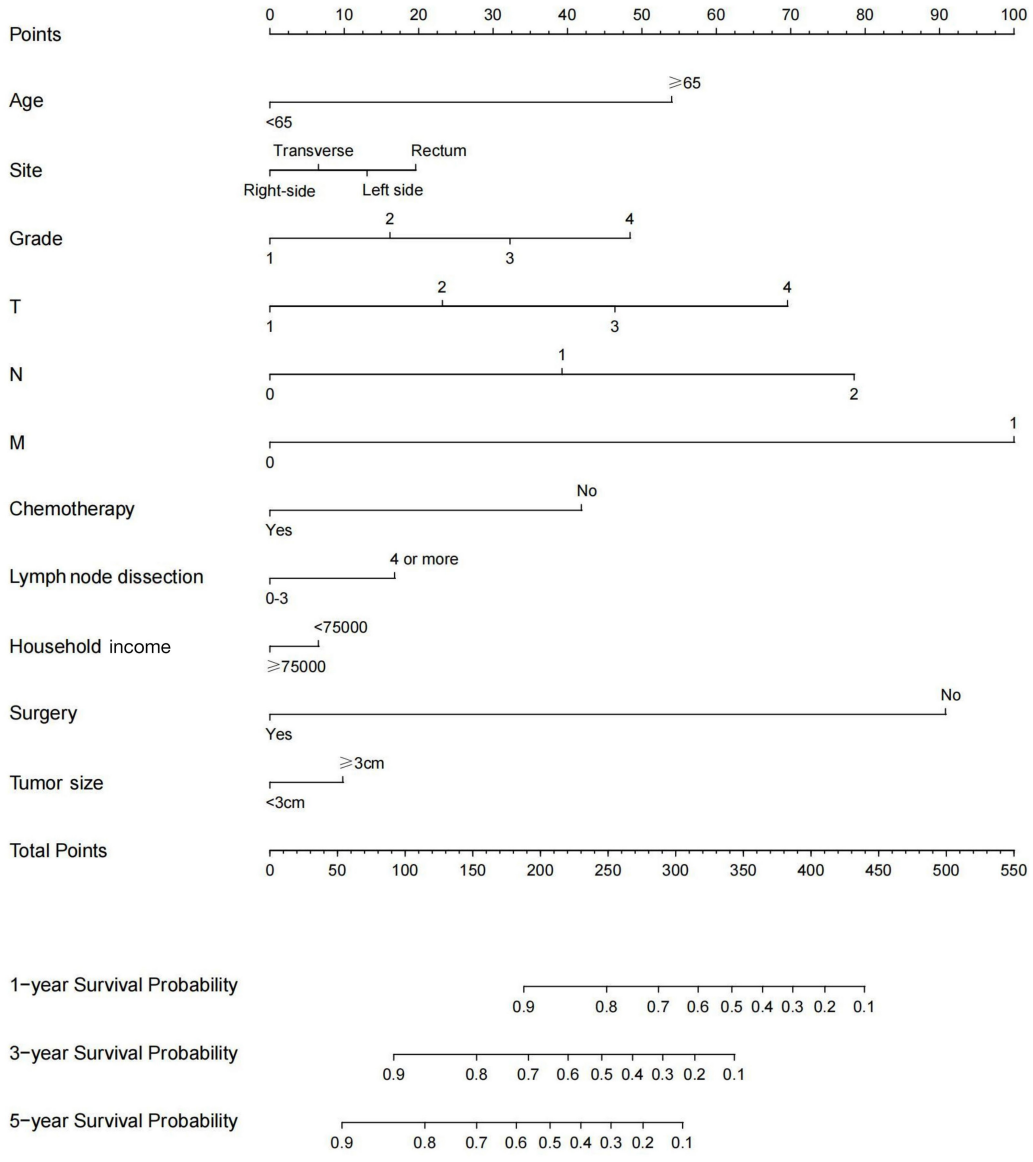
Nomogram model to predict the 1-, 3-, and 5-year overall survival of colorectal mucinous adenocarcinoma patients.

In the univariate analysis, 11 variables were associated with survival: age at diagnosis, primary site, grade, tumour size, lymph node dissection, T stage, N stage, M stage, surgery for primary site, chemotherapy, and median household income. The above variables were then included in the stepwise model, and multivariate analysis showed that all these variables were independent prognostic factors for survival (*P* < 0.05). The variance inflation factor exam suggested that multicollinearity issues did not exist (Figure S1). More details about the results of univariate and multivariate Cox regression analyses are presented in Table 2. A nomogram for predicting 1-, 3-, and 5-year survival was constructed based on the independent prognostic factors (Figure 2). As shown in the nomogram, the survival possibility of individual patients can be easily calculated by adding the scores of each variable.

**Table 1:** Demographics and pathological characteristics of enrolled colorectal mucinous adenocarcinoma patients

**Variable**	**All patients, N (%)**	**Training group, N (%)**	**Validation group, N (%)**	***P* value**
*Age (years)*				0.986
< 65	4688 (43.2)	3253 (43.2)	1435 (43.2)	
≥ 65	6158 (56.8)	4274 (56.8)	1884 (56.8)	
*Sex*				
Male	5207 (48.0)	3628 (48.2)	1579 (47.6)	0.548
Female	5639 (52.0)	3899 (51.8)	1740 (52.4)	
*Race*				0.904
White	8793 (81.1)	6106 (81.1)	2687 (81.0)	
Black	1144 (10.5)	796 (10.6)	348 (10.5)	
Other	909 (8.4)	625 (8.3)	284 (8.6)	
*Primary site*				0.958
Right-side colon	6645 (61.3)	4622 (61.4)	2023 (61.0)	
Transverse	894 (8.2)	617 (8.2)	277 (8.3)	
Left-side colon	2324 (21.4)	1604 (21.3)	720 (21.7)	
Rectum	983 (9.1)	684 (9.1)	299 (9.0)	
*Grade*				0.206
Well	1374 (12.7)	948 (12.6)	426 (12.8)	
Moderate	7171 (66.1)	5002 (66.5)	2169 (65.4)	
Poor	1832 (16.9)	1278 (17.0)	554 (16.7)	
Undifferentiated	469 (4.3)	299 (4.0)	170 (5.1)	
*Tumour size*				0.518
< 3 cm	1888 (17.4)	1322 (17.6)	566 (17.1)	
≥ 3 cm	8958 (82.6)	6205 (82.4)	2753 (82.9)	
*Lymph node dissection*				0.794
Less than 4	790 (7.3)	545 (7.2)	245 (7.4)	
4 or more	10056 (92.7)	6982 (928)	3074 (92.6)	
*T*				0.457
Tis-T1	414 (3.8)	275 (3.7)	139 (4.2)	
T2	1220 (11.2)	835 (11.1)	385 (11.6)	
T3	6048 (55.8)	4218 (56.0)	1830 (55.1)	
T4	3164 (29.2)	2199 (29.2)	965 (29.1)	
*N*				0.132
N0	5807 (53.5)	4016 (53.4)	1791 (54.0)	
N1	3076 (28.4)	2174 (28.9)	902 (27.2)	
N2	1963 (18.1)	1337 (17.8)	626 (18.8)	
*M*				0.806
M0	8962 (82.6)	6224 (82.7)	2738 (82.5)	
M1	1884 (17.4)	1303 (17.3)	581 (17.5)	
*Surgery*				0.532
No	133 (1.2)	89 (1.2)	44 (1.3)	
Yes	10713 (98.8)	7438 (98.8)	3275 (98.7)	
*Radiotherapy*				0.952
No/Unknown	9839 (90.7)	6829 (90.7)	3010 (90.7)	
Yes	1007 (9.3)	698 (9.3)	309 (9.3)	
*Chemotherapy*				0.797
No/Unknown	5983 (55.2)	4146 (55.1)	1837 (55.3)	
Yes	4863 (44.8)	3381 (44.9)	1482 (44.7)	
*Household income, $*				0.958
< 75000	7777 (71.7)	5396 (71.7)	2381 (71.7)	
≥ 75000	3069 (28.3)	2131 (28.3)	938 (28.3)

**Table 2:** Univariate and multivariate analyses of overall survival for patients in the training group

**Variable**	**Univariate analysis**		**Multivariate analysis**	
	**HR (95% CI)**	***P* value**	**HR (95% CI)**	***P* value**
*Age (years)*				
< 65	1 (Reference)	1	1 (Reference)	
≥ 65	1.596 (1.504–1.694)	< 0.001	1.884 (1.769–2.006)	< 0.001
*Sex*				NI
Male	1 (Reference)		–	
Female	1.007 (0.952–1.066)	0.803	–	
*Race*		0.209		NI
White	1 (Reference)		–	
Black	1.016 (0.927–1.114)	0.735	–	
Other	0.090 (0.819–1.015)	0.090	–	
*Primary site*		0.036		< 0.001
Right-side colon	1 (Reference)		1 (Reference)	
Transverse	1.014 (0.914–1.125)	0.796	1.028 (0.926–1.141)	0.603
Left-side colon	1.109 (1.034–1.189)	0.004	1.164 (1.085–1.250)	< 0.001
Rectum	1.019 (0.923–1.125)	0.713	1.217 (1.094–1.355)	< 0.001
*Grade*		< 0.001		< 0.001
Well	1 (Reference)		1 (Reference)	
Moderate	1.287 (1.168–1.419)	< 0.001	1.307 (1.183–1.445)	< 0.001
Poor	2.022 (1.814–2.254)	< 0.001	1.584 (1.415–1.774)	< 0.001
Undifferentiated	2.058 (1.770–2.394)	< 0.001	1.567 (1.342–1.829)	< 0.001
*Tumour size*		< 0.001		< 0.001
< 3 cm	1 (Reference)		1 (Reference)	
≥ 3 cm	1.547 (1.424–1.681)	< 0.001	1.212 (1.110–1.324)	< 0.001
*Lymph node dissection*				< 0.001
0–3	1 (Reference)		1 (Reference)	
4 or more	0.684 (0.620–0.756)	< 0.001	0.830 (0.737–0.936)	0.001
*T*		< 0.001		< 0.001
Tis-T1	1 (Reference)		1 (Reference)	
T2	0.941 (0.768–1.153)	0.556	0.972 (0.790–1.195)	0.786
T3	1.482 (1.240–1.771)	< 0.001	1.219 (1.010–1.472)	0.039
T4	2.610 (2.246–3.219)	< 0.001	1.803 (1.489–2.183)	< 0.001
*N*		< 0.001		< 0.001
N0	1 (Reference)	0	1 (Reference)	
N1	1.668 (1.559–1.784)	< 0.001	1.812 (1.682–1.952)	< 0.001
N2	2.778 (2.591–2.978)	< 0.001	2.664 (2.456–2.890)	< 0.001
*M*		< 0.001		< 0.001
M0	1 (Reference)		1 (Reference)	
M1	3.451 (3.240–3.675)	< 0.001	3.159 (2.938–3.396)	< 0.001
*Surgery*		< 0.001		< 0.001
No	1 (Reference)		1 (Reference)	
Yes	0.219 (0.182–0.264)	< 0.001	0.322 (0.258–0.402)	< 0.001
*Radiotherapy*				NI
No/Unknown	1 (Reference)		–	
Yes	1.057 (0.963–1.160)	0.242	–	
*Chemotherapy*				< 0.001
No/Unknown	1 (Reference)	0	1 (Reference)	
Yes	0.702 (0.656–0.788)	< 0.001	0.588 (0.549–0.630)	< 0.001
*Household income, $*				< 0.001
< 75000	1 (Reference)		1 (Reference)	
≥ 75000	0.904 (0.848–0.964)	0.002	0.895 (0.839–0.955)	< 0.001

NI: Not included in the multivariate survival analysis; HR: Hazard ratio; CI: Confidence interval.

### Nomogram validation

**Figure 3.**
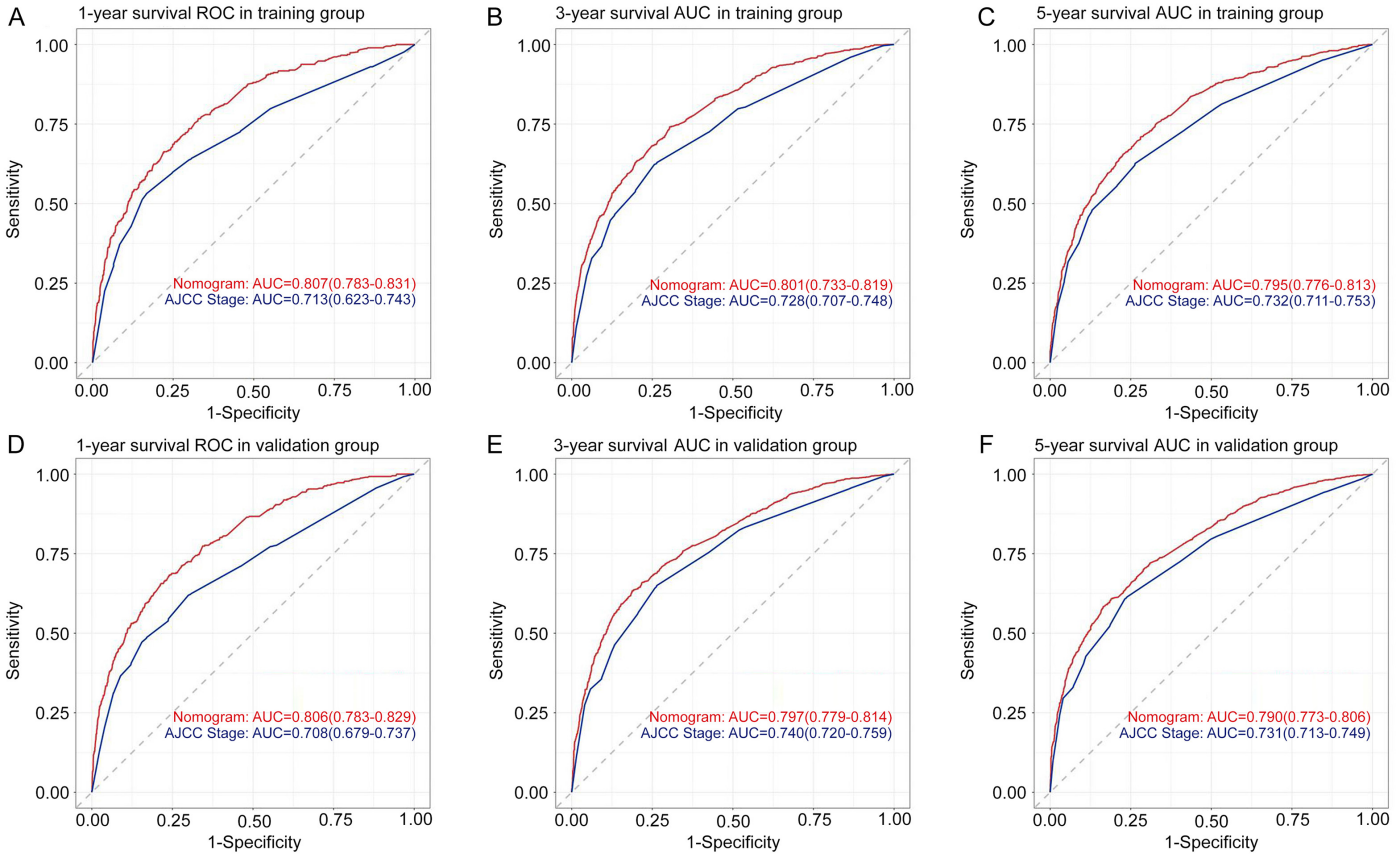
**ROC curves for predicting 1-, 3-**, **and 5-year overall survival in the training group (A)**–**(C) and validation group (D)–(F).** ROC: Receiver operating characteristics; AUC: Area under the curve; AJCC: American Joint Committee on Cancer.

**Figure 4.**
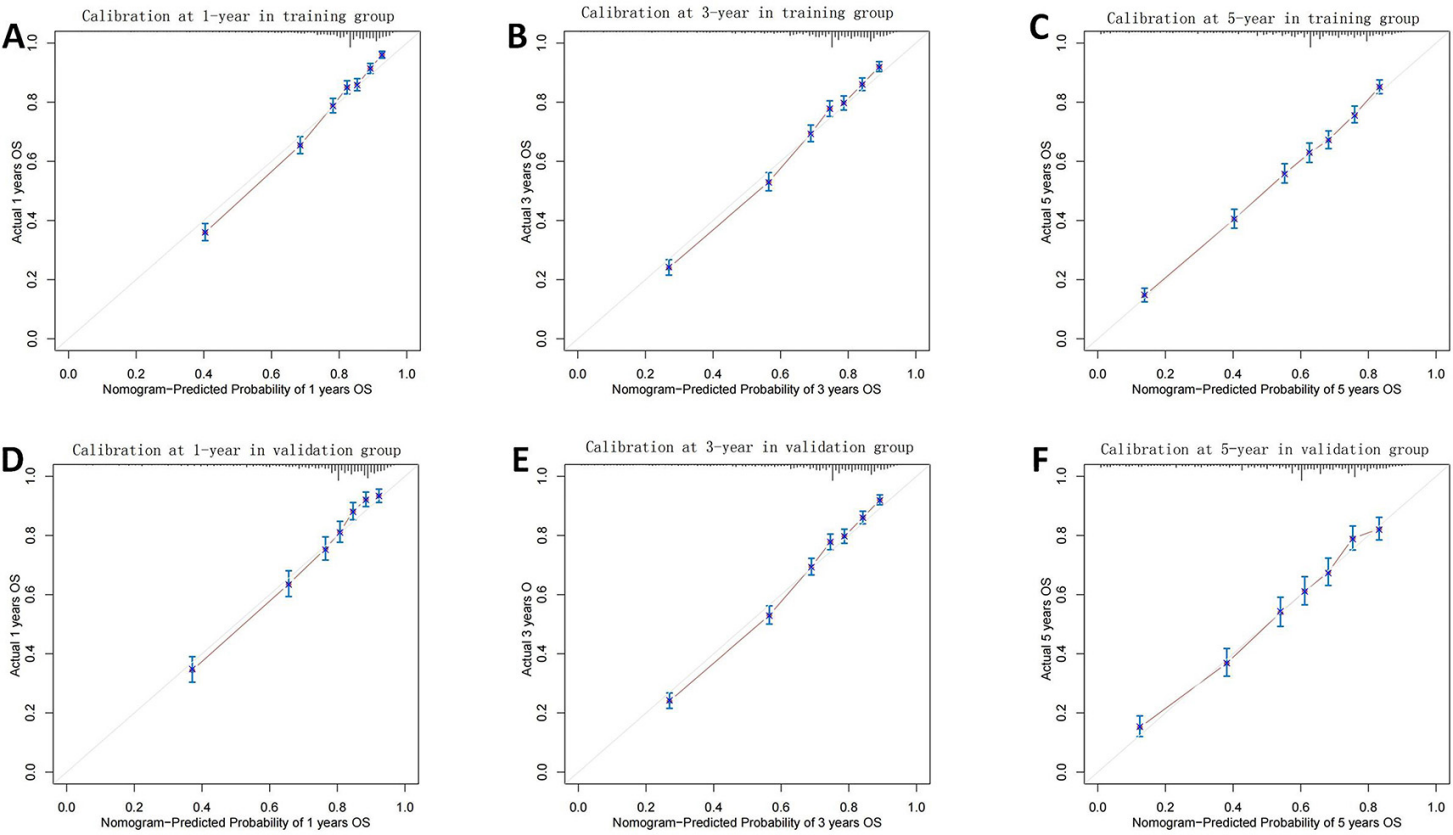
**Calibration curves for predicting 1-, 3-, and 5-year OS in the training group (A)–(C) and validation group (D)–(F)**. A 45-degree plot represented an optimal model. Vertical bars indicate 95% confidence interval. AUC: Area under the curve; AJCC: American Joint Committee on Cancer; OS: Overall survival.

The nomogram was validated internally using data from the training group and externally using data from the validation group. The C-indices of the nomogram in the training group and the validation group were 0.725 (95% confidence interval [CI] 0.716–0.734) and 0.726 (95% CI 0.713–0.739), respectively. In this study, we also calculated the C-index for both groups using the AJCC 7th TNM classification system to compare the values with our nomogram. The results showed that the prognosis-predicting ability of the nomogram was significantly better than that of the AJCC 7th TNM classification system (*P* < 0.05), as shown in Table 3. Figure 3 shows the ROC curves of the training group and the validation group. In the training group, the time-independent AUCs (tAUCs) of 1-, 3-, and 5-year OS were 0.807 (95% CI 0.783–0.831), 0.801 (95% CI 0.733–0.819), and 0.795 (95% CI 0.776–0.813), respectively. In the validation group, the tAUCs of 1-, 3-, and 5-year OS were 0.806 (95% CI 0.783–0.829), 0.797 (95% CI 0.779–0.814), and 0.790 (95% CI 0.773–0.806), respectively, which were all greater than those of the AJCC staging system. Bootstrapping with 1000 resamples in the training group exhibited a C-index of 0.723 (95% CI 0.710–0.736), which reflected consistent discrimination of the training group. The calibration curves of both the training and validation groups were close to the 45-degree line, demonstrating good consistency between the predictions and practical results. The calibration curves are shown in Figure 4.

**Table 3:** C-index for the nomogram and TNM stage system in patients with colorectal mucinous adenocarcinoma

**Classification**	**Training group**		**Validation group**	
	**C-index (95% CI)**	***P* value**	**C-index (95% CI)**	***P* value**
Nomogram	0.725 (0.716–0.734)	1 (Reference)	0.726 (0.713–0.739)	1 (Reference)
AJCC 8^th^ stage	0.674 (0.664–0.684)	< 0.001	0.679 (0.664–0.696)	< 0.001

C-index: Concordance index; CI: Confidence interval; AJCC: American Joint Committee on Cancer.

## Discussion

In this study, we analyzed 10,846 US colorectal MAC patients from the SEER database who were diagnosed during 2010–2018. After randomly dividing the patients into the training group and the validation group in a 70:30 ratio, we established an effective nomogram to predict the 1-, 3-, and 5-year OS in the training group and then internally and externally validated the discrimination and calibration of the model. Additionally, we compared the predictive capacity of our nomogram to the TNM staging system by the C-index as well as ROC values, and both presented a significant increase (*P* < 0.001), reflecting the superiority of this nomogram as a predictive tool for the prognosis of colorectal MAC patients.

MAC is a histological subtype of CRC with significant molecular differences in comparison to NMAC, for instance, overexpression of the mucin 2 and MUC5AC proteins and a high frequency of microsatellite instability (MSI-H). The relationship between molecular differences and clinical features is still undefined [4, 14, 15]. MAC has been reported to have worse clinical characteristics than NMAC, including a larger size and deeper invasion in primary lesions and higher rates of nodal metastasis and peritoneal metastasis. MAC also occurs more frequently in younger patients and females, located in the right colon and is less sensitive to radiotherapy and chemotherapy [5, 16, 17]. In our study, among all 10,846 included patients, 52% were female, the majority of the patients were initially diagnosed as having a cancer stage of T3 (56%) or T4 (29.2%), and more than half of the tumours (61.3%) occurred in the right colon, which was in agreement with the previous studies. Our study did not summarize the data of patients with peritoneal metastasis because of data deficiency. The reason why MAC tends to spread to the lymph nodes and peritoneum may be because of the pressure of mucus, which can be taken up by the lymphatic system and can spread into regional lymph nodes or push the MAC cells to the peritoneal cavity [5, 18]. The consensus is that the location of the primary tumour is significantly related to prognosis. The outcomes of patients with adenocarcinoma of the left colon were better than those of patients with adenocarcinoma of the right colon [19, 20]. In our study, the primary site was also an independent factor for prognosis, but only the comparison between cancer of the left and right colon was significantly different. The hazard ratio results indicated that the prognosis of left colon cancer is worse than that of right colon cancer. There are two possible explanations for this condition. First, appendix MAC, which was assigned to the right colon in our study, is considered to have a good prognosis [21]. Second, studies have shown that the relationship between primary location and prognosis in MAC is different from that in NMAC. One study aimed to determine the prognosis and molecular differences in MAC and NMAC in CRC and demonstrated that right colon MAC was associated with more MSI-H tumours and a similar 5-year OS rate compared with NMAC. On the other hand, the left colon and rectal MAC were related to a worse 5-year OS rate [22]. Another study retrospectively analyzed 244,794 patients from the National Cancer Database and concluded that MAC of the rectum is associated with poorer survival [23].

The TNM stages are the most important and generally acknowledged prognostic factors for patients with malignant solid tumours. Our study indicated a consistent result: TNM stages, especially the M stage, were the most valuable variables among all factors. There was another pivotal variable, surgery, which remarkably impacted the prognosis according to our nomogram. Additionally, four or more regional lymph node dissections during surgery were found to be a beneficial factor for colorectal MAC patients. These results have been proven in numerous studies and illustrate the importance of standard surgical treatment, which is the only chance for a radical cure or long-term survival [10, 24].

Whether MAC is related to poor outcomes in CRC patients is controversial. Unlike signet ring cell CRC, MAC is not identified as an independent factor for worse prognosis by the AJCC, and neither the National Comprehensive Cancer Network (NCCN) nor the European Society for Medical Oncology (ESMO) guidelines suggest disparate standard treatments particularly designed for MAC [25, 26]. Several studies indicated that no significant differences were shown between MAC and NMAC survival [27, 28]. Nevertheless, most of these studies had insufficient sample sizes, and more studies with large scales have demonstrated that MAC is an independent significant factor compared with NMAC, especially in the specific groups. Numata et al. [16] identified that MAC was associated with worse survival than NMAC in patients with stages III and IV disease. In a study including 6475 patients with stages I to III CRC, MAC was not an independent prognostic factor of disease-free survival in the entire cohort but was a significant factor in the colon subgroup analysis (*P* ═ 0.026) [5]. A meta-analysis including 44 studies also showed worse survival in colorectal MAC vs adenocarcinoma patients [29].

Despite the ambiguity in MAC prognosis, a consensus has been reached regarding the impaired response to chemotherapy. It has been observed that compared with NMAC, MAC is less responsive to neoadjuvant chemotherapy, adjuvant chemotherapy, and palliative chemotherapy, expressed by lower disease-free survival rates and survival rates [4, 30, 31]. Although our nomogram verified chemotherapy as an independent factor of prognosis, patients who underwent chemotherapy had better survival. Under these circumstances, it is in close agreement that patients with MAC could require more consideration during follow-up or even intensified adjuvant therapy.

There was a nomogram for the prognosis of colorectal MAC patients constructed by Lian et al. Compared to our nomogram study, this previous study was mainly focused on the pattern of distant metastases, and external validation was absent [32]. Our study was larger in scale and more comprehensive. We included 11 independent factors that were selected by both univariate analysis and multivariate backward stepwise Cox proportional hazard regression analysis. After excluding multicollinearity problems, a nomogram was successfully constructed. Moreover, both the C-index and ROC results demonstrated a significant advantage in prognosis compared with TNM stage classification. Additionally, the employment of our nomogram is convenient and practical in clinical work. The prognosis of each patient can be simply estimated by adding the scores of each factor and finding the corresponding possibility of the total score.

Certain limitations existed in this research. First, CRC is a heterogeneous disease, and the prognosis of CRC is affected by many factors. In addition to the characteristics mentioned in our study, clinical information was not reported or was reported incompletely in the SEER database, for instance, gene status, such as *KRAS*, *NRAS*, *BRAF* mutations, and MSI status, details of treatments, such as regimens of chemotherapy, target therapy, and immunotherapy, family history of disease, and comprehensive pathology descriptions, which may influence the effectiveness of our nomogram [33]. Further studies including these aspects are needed. Second, since this was a retrospective study and patients with incomplete data were excluded according to the study design, selection bias was inevitable. To avoid this bias, a prospective randomized controlled study should be performed. Third, data for external verification were also extracted from the SEER database, which may be less convincing. All these conditions should be considered during the application of our nomogram.

## Conclusion

In conclusion, this study constructed a practical and user-friendly nomogram based on the SEER database to predict 1-, 3-, and 5-year OS in patients with colorectal MAC. The nomogram was validated both internally and externally and demonstrated a significant advantage in predictive accuracy compared to TNM stage classification. Due to the retrospective design and the absence of more clinical and gene information, prospective studies are required in the future.

## Supplemental Data

**Figure S1.**
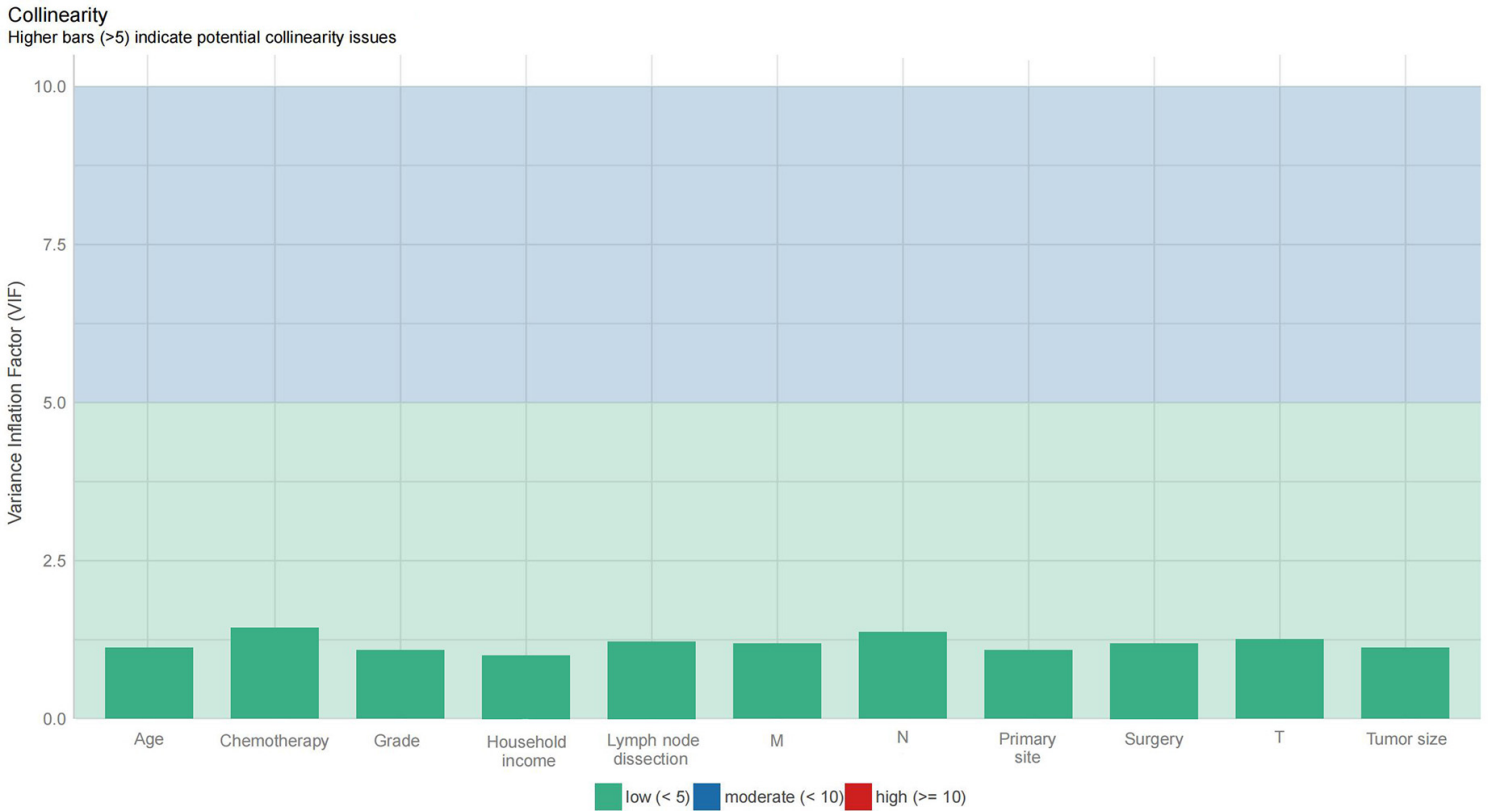
Variance inflation factor examination of included prognostic factors.
